# Effect of Sipjeondaebo-Tang on the Pharmacokinetics of S-1, an Anticancer Agent, in Rats Evaluated by Population Pharmacokinetic Modeling

**DOI:** 10.3390/molecules22091488

**Published:** 2017-09-07

**Authors:** Tae Hwan Kim, Soyoung Shin, Jeong Cheol Shin, Jürgen B. Bulitta, Kwon-Yeon Weon, Sun Dong Yoo, Gi-Young Park, Seok Won Jeong, Dong Rak Kwon, Byung Sun Min, Mi Hee Woo, Beom Soo Shin

**Affiliations:** 1 Center for Pharmacometrics and Systems Pharmacology, Department of Pharmaceutics, College of Pharmacy, University of Florida, Orlando, FL 32827, USA; taehwan.kim@cop.ufl.edu (T.H.K.); JBulitta@cop.ufl.edu (J.B.B.); 2 College of Pharmacy, Wonkwang University, Iksan, Jeonbuk 54538, Korea; shins@wku.ac.kr; 3 College of Pharmacy, Catholic University of Daegu, 13-13 Hayang-ro, Hayang-eup, Gyeongsan-si, Gyeongbuk 38430, Korea; sjc0211@gmail.com (J.C.S.); weonky@cu.ac.kr (K.-Y.W.); jswpia@gmail.com (S.W.J.); bsmin@cu.ac.kr (B.S.M.); woomh@cu.ac.kr (M.H.W.); 4 School of Pharmacy, Sungkyunkwan University, Suwon, Gyeonggi-do 16419, Korea; sdyoo@skku.ac.kr; 5 Department of Rehabilitation Medicine, School of Medicine, Catholic University of Daegu, Daegu 42472, Korea; parkgy@cu.ac.kr (G.-Y.P.); coolkwon@cu.ac.kr (D.R.K.)

**Keywords:** herbal medicine, drug-drug interaction, pharmacokinetics, 5-FU, gimeracil, Sipjeondaebo-tang

## Abstract

S-1 (TS-1^®^) is an oral fluoropyrimidine anticancer agent containing tegafur, oteracil, and gimeracil. Sipjeondaebo-tang (SDT) is a traditional oriental herbal medicine that has potential to alleviate chemotherapy-related adverse effects. The aim of the present study was to evaluate the effect of SDT on the pharmacokinetics of S-1. Sprague-Dawley rats were pretreated with a single dose or repeated doses of SDT for seven consecutive days (1200 mg/kg/day). After the completion of pretreatment with SDT, S-1 was orally administered and plasma concentrations of tegafur, its active metabolite 5-FU, and gimeracil were determined by liquid chromatography-tandem mass spectrometry (LC/MS/MS). A population pharmacokinetic model was developed to evaluate the effect of SDT on pharmacokinetics of tegafur and 5-FU. Although a single dose of SDT did not have any significant effect, the absorption rate of tegafur decreased, and the plasma levels of 5-FU reduced significantly in rats pretreated with SDT for seven days in parallel to the decreased gimeracil concentrations. Population pharmacokinetic modeling also showed the enhanced elimination of 5-FU in the SDT-pretreated group. Repeated doses of SDT may inhibit the absorption of gimeracil, an inhibitor of 5-FU metabolism, resulting in enhanced elimination of 5-FU and decrease its plasma level.

## 1. Introduction

S-1 (TS-1^®^) is a widely used oral anticancer agent for the treatment of gastric cancer, and it consists of tegafur, gimeracil (2-chloro-2,4-dihydroxypyridine), and oteracil potassium [1,2]. Tegafur is a prodrug, which is rapidly converted to cytotoxic 5-fluorouracil (5-FU), gimeracil maintains the plasma level of 5-FU by competitive inhibition of 5-FU degradation via dihydropyrimidine dehydrogenase, and oteracil potassium inhibits the phosphorylation of 5-FU in the gastrointestinal tract, thereby decreasing the toxicity of 5-FU [1,3,4]. The response rates to S-1 in patients with advanced gastric cancer was more than 40% in phase II studies [5,6,7]. S-1 has been known as an effective adjuvant treatment for patients who have undergone a gastrectomy with extended (D2) lymph node dissection for advanced gastric cancer [4]. In addition, S-1 is an effective adjuvant chemotherapy for resected pancreatic cancer compared to standard treatment with gemcitabine [8]. Thus, it is considered an important therapeutic option for the management of pancreatic cancer [9].

Since S-1 is used as postoperative adjuvant chemotherapy, it could be combined with other adjuvants including various herbal medicines to improve the quality of life in patients. Additional treatments may be required to prevent the major toxicities associated with S-1 therapy, such as hematological and gastrointestinal toxicities [10]. Herbal medicines may provide useful options to relieve chemotherapy-related toxicities and improve the conditions of patients after surgery or illness. The concurrent use of herbs and pharmaceutical drugs may mimic, augment, or oppose the pharmacokinetics or pharmacodynamics, thereby increasing or decreasing the pharmacological or toxicological effects of either constituent [11]. Although drug interactions of S-1 with certain conventional western drugs have been reported [12,13], there has been no information on drug-herb interaction between S-1 and herbal medicines. 

Sipjeondaebo-tang (SDT) is a frequently prescribed herbal medicine for the treatment of weakness after illness, including anorexia, night sweats, cold hands and feet, and anemia [14]. It has been shown that SDT is an effective antiemetic, and ameliorates cancer-induced anorexia [14,15,16,17]. In addition, SDT exhibited anticancer activities in mice [18,19] and improved cisplatin toxicity [20]. SDT consists of 10 herbs: Ginseng Radix Alba, Atractylodis Rhizoma Alba, Poria Sclerotium, Glycyrrhizae Radix, Angelicae Gigantis Radix, Cnidii Rhizoma, Rehmanniae Radix Preparata, Paeoniae Radix, Astragali Radix, and Cinnamomi Cortex [14,15]. Therefore, SDT is potentially used to improve quality of life and alleviate toxicity in patients undergoing chemotherapy. Nevertheless, little information is available on the herb-drug interaction of SDT with conventional anticancer drugs.

The objective of the present study was to evaluate the potential pharmacokinetic interaction of S-1 with the traditional herbal medicine SDT, when used in combination in postoperative patients with gastric cancer. The effects of SDT on S-1 pharmacokinetics were examined by evaluating the pharmacokinetics of S-1 administered orally in rats following repeated dosing of SDT by population pharmacokinetic modeling approach.

## 2. Results

### 2.1. Pharmacokinetics of S-1 after Pretreatment with a Single Dose of SDT

**Figure 1:**
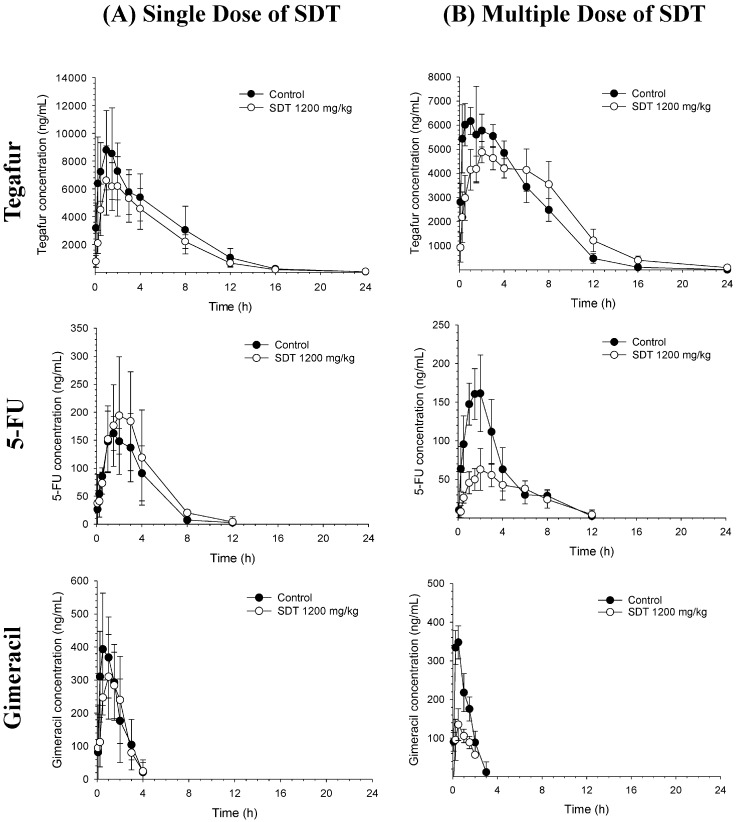
Average plasma concentration vs. time profiles of tegafur, 5-FU, and gimeracil after oral administration of S-1 to rats pretreated with 1% CMC-Na (Control, *n* = 5) or Sipjeondaebo-tang 1200 mg/kg (SDT, *n* = 5) as (**A**) a single dose or (**B**) multiple doses prior to S-1 administration.

To examine the effects of SDT on S-1 pharmacokinetics, SDT was administered as a single dose or multiple doses for seven successive days prior to S-1 administration, and S-1 pharmacokinetics was compared with that in the control (sodium carboxymethyl cellulose, CMC-Na)-pretreated group. CMC-Na is commonly used adjuvant for drugs and suspending agent which increases physical stability of suspension due to its viscosity. The average plasma concentrations vs. time profiles of tegafur, 5-FU, and gimeracil after oral administration of S-1 following SDT administration are shown in Figure 1. The average noncompartmental pharmacokinetic parameters are summarized in Table 1. Following S-1 oral administration, tegafur was rapidly absorbed, reached its maximum concentration (C_max_) within 1.5 ± 0.4 h, and disappeared from the plasma with a terminal half-life (t_1/2_) of 2.3 ± 0.7 h. As tegafur was converted to 5-FU, the T_max_ of 5-FU was 1.7 ± 0.8 h and the t_1/2_ was similar to that of tegafur (2.6 ± 2.1 h). The ratio of the area under the concentration-time curve (AUC) of 5-FU and tegafur was estimated to be 2.5 ± 0.9%. Gimeracil was also rapidly absorbed (T_max_ = 0.6 ± 0.4 h) and eliminated quickly from the plasma with a t_1/2_ of 1.2 ± 0.2 h.

No significant changes in the plasma concentration-time profiles of tegafur, 5-FU, and gimeracil were observed following single-dose SDT pretreatment compared to the control group (Figure 1A). The t_1/2_ and apparent volume of distribution during the terminal phase (V_z_) of tegafur in the SDT single-dose group were greater than that in the control group (Table 1). Nevertheless, systemic exposure to tegafur represented by AUC was not significantly affected by single dose of SDT (Table 1). The metabolic conversion of tegafur to 5-FU was also comparable between the two groups.

### 2.2. Pharmacokinetics of S-1 after Pretreatment with Multiple Doses of SDT

Overall pharmacokinetics of tegafur and 5-FU obtained after single and multiple dose of control vehicle (1% CMC-Na) were comparable. However, the AUC of gimeracil was lower in rats treated with multiple dose of 1% CMC-Na than that in rats treated with single dose of 1% CMC-Na.

Unlike pretreatment with single dose, pretreatment with multiple doses of SDT prior to S-1 significantly delayed the absorption of tegafur and decreased the plasma levels of 5-FU and gimeracil (Figure 1B and Table 1). The T_max_ of tegafur in groups administered repeated doses of SDT was 3.2 ± 1.6 h (vs. 1.2 ± 0.7 in control group), and the C_max_ of tegafur was reduced to 72.6% compared to that in control group; however, AUC remained unchanged. Although the AUC of tegafur was not significantly altered in groups treated with repeated doses of SDT, the plasma levels of its active metabolite 5-FU reduced markedly. The AUC ratio of 5-FU to tegafur was reduced to 55.6% compared to that in the control. Compared to the control group, groups treated with repeated doses of SDT showed a substantial decrease in plasma concentration of gimeracil, with a C_max_ and AUC of 40.9% and 47.0%, respectively.

### 2.3. Population Pharmacokinetic (POP-PK) Modeling

**Figure 2:**
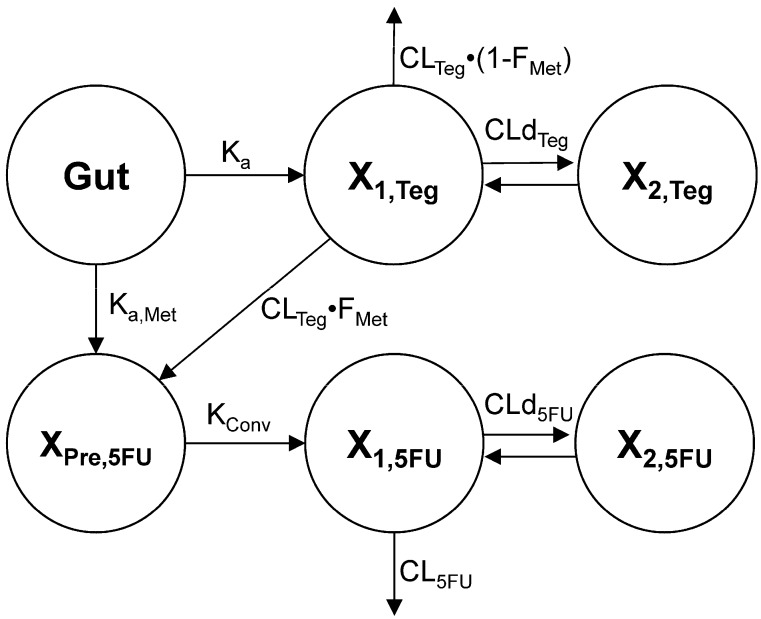
Structural model for the absorption and disposition of tegafur and its active metabolite, 5-FU in rats.

**Figure 3:**
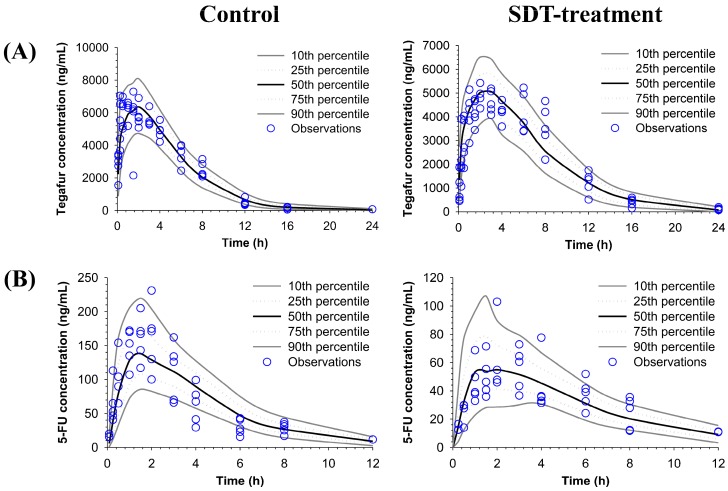
Visual predictive check plots of the population pharmacokinetic model for (**A**) tegafur and (**B**) 5-FU. The observed plasma concentration of tegafur and 5-FU in control and pretreated with repeated doses of SDT group (open circles) are shown with the lines representing the 10th, 25th, 50th, 75th and 90th percentiles of the population predictions.

To describe the oral absorption and metabolic conversion of tegafur to its active metabolite 5-FU, the plasma concentration profiles of tegafur and 5-FU obtained after oral administration of S-1 (5 mg/kg as tegafur) were simultaneously fitted to the POP-PK model (Figure 2). The visual predictive checks by comparing predicted vs. observed plots showed an excellent predictive performance of the POP-PK model (Figure 3). The POP-PK parameter estimates for tegafur and 5-FU of the control and SDT pretreatment group are shown in Table 2.

To determine the effect of SDT multiple dose pretreatment, the oral absorption rate of tegafur (K_a_), metabolic conversion rate of tegafur to 5-FU precursor in the gut and liver by first pass metabolism (K_a,Met_), and the clearance of 5-FU (CL_5FU_) were separately estimated for control and SDT pretreated group. Consistent with the extended T_max_ and reduced C_max_ obtained by the noncompartmental analysis, the K_a_ of the SDT pretreatment group was slower than that of the control group. The metabolic conversion rate of tegafur to 5-FU precursor in the gut and liver (K_a,Met_) was also slower in the SDT pretreated group than that in the control group. The clearance of 5-FU (CL_5FU_) in the SDT pretreatment group was 1.68-fold higher than that in the control group, which is comparable to the decrease in 5-FU AUC in the SDT pretreatment group.

## 3. Discussion

This study evaluated the potential drug-herb interaction between the anticancer agent S-1 and traditional herbal medicine SDT for alleviating chemotherapy-related adverse effects. The effects of SDT on S-1 pharmacokinetics were examined after pretreatment of rats with SDT. The plasma concentrations of 5-FU and its precursor and metabolic inhibitor, tegafur and gimeracil respectively, were determined to examine the pharmacokinetics of S-1. The overall effects of SDT on the pharmacokinetics of S-1 were examined by noncompartmental analysis and the alteration in the absorption of tegafur as well as metabolism of 5-FU were further assessed by population pharmacokinetic modeling approach.

Pharmacokinetics of tegafur and gimeracil are known to be the main determinants of the pharmacological activity of 5-FU after S-1 administration [21]. In addition, the plasma level of 5-FU is affected by formation of 5-FU by means of the metabolic conversion of tegafur to 5-FU and degradation of 5-FU. The present results clearly showed that repeated dosing of SDT substantially decreased the plasma levels of 5-FU in parallel with the reduced AUC of gimeracil, the metabolic inhibitor of 5-FU. On the other hand, the precursor of 5-FU, tegafur plasma concentrations were not significantly affected by SDT administration. 

Since 5-FU is formed via metabolic activation of tegafur and its elimination is inhibited by gimeracil, the lower plasma concentration of 5-FU may be associated with either the reduced formation of 5-FU due to the decreased metabolism of tegafur or increased metabolism of 5-FU due to the reduced absorption of gimeracil. The formation and metabolism of 5-FU are mediated by different enzymatic systems. The formation of 5-FU from tegafur is mediated by CYP1A and CYP3A in rats [22] and by CYP2A6 in humans via the intermediate 5’-hydroxytegafur [23], while the degradation of 5-FU is mainly mediated by dihydropyrimidine dehydrogenase [24]. Either the reduced metabolism of tegafur or enhanced metabolism of 5-FU may be reflected to its own terminal half-life in the plasma concentration vs. time profile. 

However, our data indicated that the terminal half-life of 5-FU was not significantly affected by SDT multiple dosing despite a decrease of 5-FU plasma level. The elimination half-life of 5-FU is known to be short (t_1/2_ < 20 min) [25,26,27]. Our preliminary study also confirmed the short t_1/2_ of 5-FU (19.3 ± 7.5 min) following intravenous dose of 5-FU at 10 mg/kg. After S-1 administration, however, the average t_1/2_ of 5-FU was found to be 2.2–3.1 h, which is similar to that of tegafur (2.2–3.5 h) (Table 1). As indicated by the parallel declines of plasma tegafur and 5-FU and their comparable elimination half-lives, the pharmacokinetics of 5-FU is likely formation-rate limited, in which the terminal phase of the plasma concentration-time profile is determined by its formation. Thus, the terminal phase of the plasma concentration-time profile of 5-FU following S-1 administration may not be governed by 5-FU elimination but by the slower conversion from tegafur to 5-FU, which is so-called flip-flop kinetics. Therefore, the enhanced degradation of 5-FU due to reduced gimeracil activity in SDT-treated rats may not be reflected by the terminal phase of the 5-FU plasma concentration-time profile. 

The decrease in the plasma levels of 5-FU due to the increased elimination by SDT multiple dosing was further assessed by the population pharmacokinetic modeling. The estimated clearance of 5-FU (CL_5FU_) in the SDT pretreatment group was greater than that in the control group (Table 2). The 1.68-fold increase of estimated 5-FU clearance by SDT treatment was comparable to the extent of the decreases in the AUC_all_ and AUC_inf_ of 5-FU by 0.57-fold (Table 1). Although the absorption rate (K_a_) and the metabolic rate of tegafur to 5’-hydroxytegafur, the 5-FU precursor in the gut (K_a,Met_) were also reduced by SDT pretreatment, they may not contribute to the overall 5-FU plasma AUC level. Thus, the decreased gimeracil plasma level may lead to the increased elimination of 5-FU and decreased plasma 5-FU concentrations after SDT multiple dosing. 

Mechanisms responsible for the reduced gimeracil plasma concentrations after SDT multiple dosing require further investigation. Multiple doses of SDT may affect gimeracil plasma levels via interactions mediated by metabolic enzymes or transporters. However, gimeracil is mainly excreted into urine as an unchanged form and metabolism of gimeracil is minimal. Gimeracil did not show any significant inhibitory effects or induction on various CYP isoenzymes [28]. The most important factor that affects plasma gimeracil levels is known to be renal function. Moreover, our data indicated that the C_max_ and AUC of gimeracil significantly decreased following SDT multiple dosing without changes in the elimination t_1/2_ (Table 1). Therefore, the decrease in the gimeracil plasma concentrations may be associated with its reduced absorption not with its elimination. Further studies are required to elucidate the exact mechanisms involving any transporters or enzymes in the gimeracil absorption altered by SDT multiple dosing.

It is also possible that the decreased plasma 5-FU concentrations were in part attributed to the reduced formation of 5-FU, i.e., metabolic activation of tegafur in SDT-pretreated group by various herb constituents of SDT. Modulation of metabolism via CYP enzymes is one of the common mechanisms of drug interactions. Various dietary supplements including grapefruit juice, 8-methoxypsoralen in plant species, and soy-derived products have been shown to be an inhibitor of CYP2A6 [29,30,31]. The alteration of tegafur metabolism may be reflected to the terminal half-life of tegafur as well as the that of 5-FU because of the flip-flop kinetics. However, either the half-life of tegafur or the 5-FU was not altered by SDT multiple dosing. The comparable elimination half-lives of tegafur and 5-FU suggest that the metabolism of tegafur, that is, suppression of 5-FU formation may not be the cause of the decreased 5-FU plasma concentrations by SDT multiple dosing.

Our data also indicated that gimeracil AUC values were lower after multiple doses of 1% CMC-Na, i.e., control treatment, compared to the single dose of 1% CMC-Na (Table 1). Since the single dose and multiple dose studies were conducted separately at different times, there might be period effect which may in part contribute to the different AUCs of gimeracil in control groups. It is also possible that multiple doses of CMC-Na may affect the pharmacokinetics of gimeracil. Although it is commonly used adjuvants for drugs, there has been a report on interaction of CMC-Na with other drugs. For example, concurrent administration of CMC-Na with 1-deoxynojirimycin (DNJ) resulted in significant decrease in AUC of DNJ [32].

In summary, the potential pharmacokinetic interaction of an anticancer agent (S-1) and oriental medicine (SDT) for the treatment of gastric adverse events was evaluated. Plasma concentrations of 5-FU was significantly decreased following repeated dosing of SDT, which is likely due to the enhanced degradation of 5-FU resulting from the reduced absorption of gimeracil. The clinical significance of the altered 5-FU plasma concentrations by SDT would be evaluated in further studies.

## 4. Materials and Methods

### 4.1. Materials

Tegafur, 5-FU, gimeracil, and potassium oxonate were purchased from Tokyo Chemical Industry Co., Ltd. (Tokyo, Japan). SDT was obtained from Hankook Shinyak Corp. (Nonsan, Chungnam, Korea). Sodium carboxymethyl cellulose (CMC-Na) and methyl cellulose (MC) were obtained from Junsei Chemical (Tokyo, Japan). Acetonitrile and distilled water (all HPLC grades) were purchased from J.T. Baker, Inc. (Phillipsburg, NJ, USA), and formic acid was obtained from Aldrich Chemical Co. (Milwaukee, WI, USA).

### 4.2. Animal Study

The animal study was approved by the Ethics Committee for the Treatment of Laboratory Animals at Catholic University of Daegu (IACUC-2013-025) and conducted by following the standard operating procedures (SOPs). Male Sprague–Dawley rats (8 weeks, 280–300 g; Hyochang Science, Daegu, Korea) were kept in plastic cages and provided free access to standard diet (Daejong, Seoul, Korea). The animals were maintained at a temperature of 23 ± 2 °C with a 12 h/12 h light-dark cycle and relative humidity of 50 ± 10%. 

To examine the effect of SDT on S-1 pharmacokinetics, SDT suspended in 10% CMC-Na was orally administered to rats as a single dose or repeated doses for seven consecutive days. For the single-dose study, SDT (1200 mg/kg) or 1% CMC-Na (control) was orally administered. After 1 min of SDT administration, S-1 as a mixture of 5 mg/kg tegafur, 1.45 mg/kg gimeracil, and 4.9 mg/kg oteracil potassium was dissolved in 7.5% dimethyl sulfoxide (DMSO) and orally administered by oral gavage. For the multiple doses study, SDT (1200 mg/kg) or 1% CMC-Na (control) was dosed every day for seven days and S-1 was administered by oral gavage after 1 min of the last dose of SDT. The dose of SDT was selected based on its human clinical dose. The clinical dose of SDT is 9 g/day, which corresponds to 128.6 mg/kg/day for a patient weighing 70 kg. Considering the faster metabolic process of rats compared to humans, an SDT dose approximately 10-fold higher (1200 mg/kg) than that of the human dose was administered in rats. The animals were fasted for 12 h before the S-1 dose. Following oral administration of S-1, approximately 400 µL of the venous blood samples were collected at predose, 15, 30 min, 1, 1.5, 2, 3, 4, 8, 12, and 24 h postdose from the jugular vein. Plasma samples were harvested by centrifugation of the blood samples at 16,060× *g* for 10 min and stored at −80 °C until analysis.

### 4.3. LC/MS/MS

Tegafur, 5-FU, and gimeracil concentrations were simultaneously determined by a liquid chromatography-tandem mass spectrometry (LC/MS/MS) assay. Chlorothalidone (50 µL), the internal standard (IS) working solution (100 ng/mL in acetonitrile), and 150 µL of acetonitrile as a precipitation solvent were added to 50 µL of plasma samples. The mixture was vigorously vortexed for 1 min and centrifuged for 10 min at 16,060× *g*. The supernatant (100 µL) was mixed with the same volume of distilled water, and 10 µL of the final mixture was injected onto the LC/MS/MS.

The LC/MS/MS system consisted of API 4000 triple quadrupole mass spectrometer (AB MDS Sciex, Toronto, ON, Canada) coupled with an Agilent 1100 HPLC system (Agilent, Santa Clara, CA, USA). The plasma samples were separated on a SynergiTM Fusion-RP C_18_ Column (100 × 2.0 mm i.d., 2.5 µm) and SecurityGuard Guard Cartridge (Phenomenex, Torrence, CA, USA). The mobile phase comprised a mixture of acetonitrile and 0.05% formic acid (35:65 *v/v*). The flow rate was set at 0.2 mL/min, and the column oven temperature was 30 °C. The electrospray ionization (ESI) source was operated in positive mode for tegafur and negative mode for 5-FU, gimeracil, and the IS. The curtain and turbo-gas (all nitrogen) were set at 6 and 20 psi, respectively. The mass spectrometer was operated in multiple reaction monitoring (MRM) mode with a dwell time of 300 ms per MRM channel. The selected MRM transition was *m/z* 201.1→131.0 for tegafur, *m*/*z* 128.9→42.1 for 5-FU, *m*/*z* 143.9→100.1 for gimeracil, and *m/z* 336.9→189.6 for IS. The collision energy was set at 13, −30, −22, and −26 V for tegafur, 5-FU, gimeracil, and IS, respectively. Analyst 1.4 software (AB MSD Sciex, Toronto, ON, Canada) was used for data acquisition. 

The assay was fully validated by using matrix-matched quality control samples and the lower limit of quantification (LLOQ) for tegafur, 5-FU, and gimeracil was 50, 10, and 50 ng/mL, respectively. The intra- and inter-day accuracies were 94.6–106.1% for tegafur, 96.8–112.5% for 5-FU, and 89.3–113.9% for gimeracil. The precision of tegafur, 5-FU, and gimeracil was within 12.8%, 13.4% and 11.8%, respectively.

### 4.4. Noncompartmental Analysis

The pharmacokinetic parameters were determined by noncompartmental analysis using Phoenix^®^ WinNonlin^®^ 6.4 (Certara, L.P., Princeton, NJ, USA). These parameters included terminal half-life (t_1/2_), the area under the plasma concentration-time curve from time zero to the last observation time point (AUC_all_) and infinity (AUC_inf_), V_z_/F, and systemic clearance (CL/F). The peak plasma concentration (C_max_) and the time to reach C_max_ (T_max_) were read directly from the observations.

### 4.5. Population Pharmacokinetic Modeling

The population pharmacokinetic model was developed to capture the absorption and disposition of tegafur and its active metabolite 5-FU. We considered a model with two compartments for both tegafur and 5-FU. The absorption process after oral administration of tegafur and formation of the 5-FU precursor were described by first order rate constants, K_a_ and K_a,Met_, respectively. Since the absorption of tegafur was found to be slower by SDT pretreatment in the noncompartmental analysis, the K_a_ and K_a,Met_ were estimated separately for control and SDT pretreatment group. The differential equation for the amount of tegafur in the absorption site (X_gut_) was:

(1) $$\frac{{\mathrm{dX}}_{\;\mathrm{gut}}}{\mathrm{dt}}\;=\;-(K_{a}\;+K_{a,\;\mathrm{Met}})\cdot \;X_{\;\mathrm{gut}}$$

The central compartment received tegafur from the gut compartment. The differential equation for the amount of tegafur in the central compartment (X_1,Teg_) contained terms for the drug transfer into and from the peripheral compartment and drug elimination. The differential equation for the amount of tegafur in the central compartment (X_1,Teg_) was (initial condition: 0):

(2) $$\frac{{\mathrm{dX}}_{\;1,\;\mathrm{Teg}}}{\mathrm{dt}}\;=\;K_{a}\;\cdot \;X_{\mathrm{gut}}\;-\;({\mathrm{CL}}_{\;\mathrm{Teg}}+{\mathrm{CLd}}_{\;\mathrm{Teg}})\;\cdot \;C_{1,\;\mathrm{Teg}}\;+\;{\mathrm{CLd}}_{\;\mathrm{Teg}}\;\cdot \;C_{2,\;\mathrm{Teg}}$$

where C_1,tag_ and C_2,tag_ represent tegafur concentration in their respective compartment and CL_Teg_ and CLd_Teg_ are the systemic and distribution clearances of tegafur, respectively. The differential equation for the amount of tegafur in the peripheral compartment (X_P,Teg_) was (initial condition: 0):

(3) $$\frac{{\mathrm{dX}}_{2,\;\mathrm{Teg}}}{\mathrm{dt}}\;=\;{\mathrm{CLd}}_{\mathrm{Teg}}\;\cdot \;C_{1,\;\mathrm{Teg}}\;-\;{\mathrm{CLd}}_{\mathrm{Teg}}\;\cdot \;C_{2,\;\mathrm{Teg}}$$

Tegafur is metabolized to 5’-hydroxytegafur, the precursor of 5-FU (X_Pre,5FU_) in the gut and in the central compartment and converted to 5-FU with a first order rate constant, K_Conv_ (initial condition: 0):

(4) $$\frac{{\mathrm{dX}}_{\;\mathrm{Pre},5\mathrm{FU}}}{\mathrm{dt}}\;=\;K_{a,\;\mathrm{Met}}\;\cdot \;X_{\;\mathrm{gut}}\;+\;{\mathrm{CL}}_{\;\mathrm{Teg}}\;\cdot \;F_{\mathrm{Met}}\;\cdot \;C_{1,\;\mathrm{Teg}}\;-\;K_{\mathrm{Conv}}\;\cdot \;X_{\;\mathrm{Pre},5\mathrm{FU}}$$

F_Met_ is a fraction of tegafur in the central compartment which enters the metabolic pathway to generate 5-FU precursor, which was estimated separately for control and SDT pretreated rats to determine the effect of SDT pretreatment. 5-FU is produced from the precursor, distributed to the peripheral compartment, and eliminated from the central compartment. The differential equations for the amount of 5-FU in the central and peripheral compartment were (initial conditions: 0):

(5) $$\frac{{\mathrm{dX}}_{\;1,5\mathrm{FU}}}{\mathrm{dt}}\;=\;K_{\;\mathrm{Conv}}\;\cdot \;X_{\;\mathrm{Pre},5\mathrm{FU}}\;-\;({\mathrm{CL}}_{\;5\mathrm{FU}}\;+\;{\mathrm{CLd}}_{\;5\mathrm{FU}})\;\cdot \;C_{\;1,5\mathrm{FU}}\;+\;{\mathrm{CLd}}_{\;5\mathrm{FU}}\;\cdot \;C_{\;2,5\mathrm{FU}}$$

(6) $$\frac{{\mathrm{dX}}_{2,5\mathrm{FU}}}{\mathrm{dt}}\;=\;{\mathrm{CLd}}_{5\mathrm{FU}}\;\cdot \;C_{1,5\mathrm{FU}}\;-\;{\mathrm{CLd}}_{5\mathrm{FU}}\;\cdot \;C_{2,5\mathrm{FU}}$$

where C_1,5FU_ and C_2,5FU_ represent 5-FU concentrations in their respective compartment and CL_5FU_ and CLd_5FU_ are the systemic and distribution clearances of 5-FU, respectively.

All plasma concentrations were simultaneously fitted by the population pharmacokinetic (POP-PK) modeling. The plasma concentration profiles of tegafur and 5-FU obtained after oral administration of S-1 (5 mg/kg as tegafur) and also the plasma concentration profile of 5-FU after intravenous injection of 5-FU at a dose of 10 mg/kg obtained in a previous study was also fitted. The importance sampling version of the Monte Carlo Parametric Expectation Maximization (MC-PEM) algorithm in the parallelized S-ADAPT software (version 1.57) [33] supported by the SADAPT-TRAN facilitator [34,35] was utilized for modeling. Log-normal distribution was used to describe the between subject variability (BSV) for each parameter. Residual model with additive and proportional error was used for tegafur and 5-FU concentrations [34]. The goodness of fit was assessed by visual inspection of the observed and fitted concentrations, the objective function, plausibility of parameter estimates, standard diagnostic plots, the normalized prediction distribution error (NPDE) [36], and visual predictive checks (VPCs) [37].

### 4.6. Statistical Analysis

The obtained parameters were compared by unpaired *t*-test between the two means for unpaired data. *p* values < 0.05 were considered statistically significant.

## Figures and Tables

**Table 1:** Noncompartmental pharmacokinetic parameters of tegafur, 5-FU, and gimeracil after oral administration of S-1 in rats pretreated with single dose or multiple doses of 1% CMC-Na (Control, *n* = 5) or Sipjeondaebo-tang 1200 mg/kg (SDT, *n* = 5).

	Parameter	Single Dose		Multiple Dose	
		Control (*n* = 5)	SDT (*n* = 5)	Control (*n* = 5)	SDT (*n* = 5)
Tegafur	t_1/2_ (h)	2.3 ± 0.7	3.5 ± 0.7 *	2.2 ± 1.4	3.3 ± 0.8
	T_max_ (h)	1.5 ± 0.4	1.3 ± 0.4	1.2 ± 0.7	3.2 ± 1.6 *
	C_max_ (ng/mL)	9328.0 ± 3099.3	7202.0 ± 2374.7	6828.0 ± 384.0	4960.0 ± 431.9 *
	AUC_all_ (ng·h/mL)	55,372.7 ± 20215.9	42,705.1 ± 11087.4	43,496.9 ± 4673.0	46,842.4 ± 8127.5
	AUC_inf_ (ng·h/mL)	55,712.7 ± 20247.5	42,945.5 ± 11033.1	43,748.1 ± 4835.6	47,461.3 ± 8163.9
	CL/F (mL/min/kg)	1.7 ± 0.8	2.1 ± 0.6	1.9 ± 0.2	1.8 ± 0.3
	V_z_/F (L/kg)	0.3 ± 0.1	0.6 ± 0.2 *	0.4 ± 0.2	0.5 ± 0.1
5-FU	t_1/2_ (h)	2.6 ± 2.1	2.2 ± 1.0	3.0 ± 0.9	3.1 ± 0.8
	T_max_ (h)	1.7 ± 0.8	1.7 ± 1.1	1.8 ± 0.3	2.4 ± 0.5
	C_max_ (ng/mL)	178.2 ± 42.0	209.5 ± 94.9	172.2 ± 40.4	64.3 ± 23.0 *
	AUC_all_ (ng·h/mL)	613.7 ± 233.4	892.4 ± 439.7	639.3 ± 190	362.7 ± 96.2 *
	AUC_inf_ (ng·h/mL)	924.9 ± 537.6	955.8 ± 438.9	750.2 ± 145.8	429.6 ± 83.2 *
	AUC_meta_/AUC_parent_ (%)	2.5 ± 0.9	3.3 ± 1.0	2.7 ± 0.7	1.5 ± 0.5 *
Gimeracil	t_1/2_ (h)	1.2 ± 0.2	0.9 ± 0.3	0.7 ± 0.1	0.8 ± 0.2
	T_max_ (h)	0.6 ± 0.4	0.8 ± 0.8	0.5 ± 0.0	0.7 ± 0.5
	C_max_ (ng/mL)	422.2 ± 136.5	358 ± 121.4	347.8 ± 42.7	142.3 ± 41.8 *
	AUC_all_ (ng·h/mL)	768.3 ± 312.9	678.7 ± 190.9	449.5 ± 74.2	180.2 ± 41.5 *
	AUC_inf_ (ng·h/mL)	984.8 ± 335.4	758.7 ± 186.7	526.6 ± 85.4	247.4 ± 57.4 *
	CL/F (mL/min/kg)	26.8 ± 8.8	33.7 ± 9.4	46.9 ± 7.8	101.9 ± 22.6 *
	V_z_/F (L/kg)	2.9 ± 1.3	2.6 ± 1.4	2.8 ± 0.4	6.8 ± 0.9 *

* *p* < 0.05 vs. Control.

**Table 2:** Population pharmacokinetic parameter estimates of tegafur and its active metabolite, 5-FU.

Parameter	Symbol	Unit	Population Mean (BSV)
Absorption rate constant for tegafur in control group	K_a,Con_	1/h	0.296 (0.0101)
Absorption rate constant for tegafur in SDT pretreatment group	K_a,Pretre_	1/h	0.197 (0.0125)
Formation rate constant of 5-FU precursor from gut compartment in control group	K_a,Met,Con_	1/h	0.122 (0.308)
Formation rate constant of 5-FU precursor from gut compartment in SDT pretreatment group	K_a,Met,Pretre_	1/h	0.0595 (0.655)
Formation rate constant of 5-FU from 5-FU precursor	K_Conv_	1/h	2.88 (0.747)
Clearance for tegafur	CL_Teg_/F	L/h/kg	0.0813 (0.00221)
Fraction of 5-FU clearance for 5-FU precursor formation	F_Met_	-	0.342 (0.0162)
Clearance for 5-FU in control group	CL_5FU,Con_/F	L/h/kg	3.52 (0.163)
Clearance for 5-FU in SDT pretreatment group	CL_5FU,Pretre_/F	L/h/kg	5.93 (0.025)
Distribution clearance for tegafur	CLd_Teg_/F	L/h/kg	0.184 (0.105)
Distribution clearance for 5-FU	CLd_5FU_/F	L/h/kg	1.87 (0.168)
Central volume of distribution for tegafur	V_1,Teg_/F	L/kg	0.0464 (0.729)
Central volume of distribution for 5-FU	V_1,5FU_/F	L/kg	0.623 (0.291)
Peripheral volume of distribution for tegafur	V_2,Teg_/F	L/kg	0.137 (0.0141)
Peripheral volume of distribution for 5-FU	V_2,5FU_/F	L/kg	0.294 (0.184)
